# Supplementary material for: Research on the semantic segmentation of Thangka images via an improved PIDNet
Source: PLoS One. 2026 May 14;21(5):e0332164. doi: 10.1371/journal.pone.0332164 (PMC13175359; doi:10.1371/journal.pone.0332164)
Supplement: Attachment — Submitted filename: PONE-D-25-46097_review.pdf [file pone.0332164.s002.pdf]

General Comment to Authors

The Authors present a new network structure to segment the motifs of Thangka images, in the context of preservation of the artworks that may benefit in case of digitalization, archiving and restoration efforts.

The presented concept and methodology look solid in their description and performances. The Authors seem to significantly improve the current state of the art, balancing accuracy and complexity.

However, this work requires major revisions as it lacks form.

First of all, it is unacceptable to submit a manuscript with the presence of unrelated text (lines 118-125). This makes me wonder about the level of attention the Authors devoted to pull together this manuscript.

Then, it is also unacceptable for a scientific publication to not have figure and tables captions.

The last two major form points regard the usage of acronyms and their shuffled definitions throughout the manuscript, and the structure of the results section which contains many elements that instead belong in the methods section.

Please find a detailed list of review points below

Review Points

ABSTRACT

1. Line 17: Perhaps spell out ECA-Pag and LGFM modules
2. Line 19-20: The presented figures are far from being conventional, especially in the field of conservation/preservation. Also, one of the metrics was developed in the work, so it is not possible to assume the readers know what that is.

INTRODUCTION

3. Line 42-43: why is it important to stress real-time performance in a conservation science setting?
4. Figure 1 and all the other figures: it is unacceptable to have no captions.
5. Line 72: missing reference for UNetFormer.
6. Line 96: LGFM never defined

METHODS

7. Line 118-125: it is unacceptable to submit a manuscript like this
8. Line 127: PID should have been spelled out earlier
9. Line 132-133: you already have explained the three paths at lines 134-135
10. Line 147-151: unnecessary in the methodology section
11. There is major confusion regarding what constitutes a result and what is methodology. Sections 3.1 through 3.4 do not belong in the Results section

12. Table 1: not acceptable to present a table like this. Also valid for all tables, like for the figures, there should be captions.
13. Line 317: the fact that you introduce the mB-Fscore as a new metrics should be stated in the abstract, introduction and metods
14. The example on the Cityscape dataset comes a little bit out of nowhere, if your goal is to demonstrate the generalization abilities of your approach, why not using more datasets?
15. Line 336: The spell out of FPS comes too late after FPS has been mentioned a few times
