# Supplementary material for: Research on the semantic segmentation of Thangka images via an improved PIDNet
Source: PLoS One. 2026 May 14;21(5):e0332164. doi: 10.1371/journal.pone.0332164 (PMC13175359; doi:10.1371/journal.pone.0332164)
Supplement: Attachment — Submitted filename: Response to Reviewers.docx [file pone.0332164.s004.docx]

**Manuscript Number**: PONE-D-25-46097
**Title**: Research on the Semantic Segmentation of Thangka Images via an Improved PIDNet
**Authors**: Jiao Wu, Tiejun Wang*, Xiaoyan Hu, LingMei Tao, Tianjiao Duan, Yanjiao Wei

Dear Editor Dr. Baohua Wen and Reviewers,

We would like to thank you for the opportunity to revise our manuscript and for the constructive comments provided by the reviewers. We have carefully studied the feedback and have made significant revisions to the manuscript to address all concerns.

Specifically, we have restructured the "Methods" and "Results" sections to improve logical flow, added statistical analysis (mean ± standard deviation) from 5 independent runs to ensure rigor, and corrected all formatting and citation issues.

Below is a point-by-point response to the editors’ and reviewers’ comments.

### **Response to Journal Requirements**

**1. Style and Formatting:** We have carefully formatted the manuscript according to PLOS ONE’s style templates. We checked file naming, heading styles, and figure citations to ensure compliance.

**2. Methods and Data Source:** We have added details about the dataset construction in Section 2.6. We explicitly state that all data collection and usage complied with the terms and conditions of their sources.

**3. Code Sharing:** The implementation code for the proposed method is currently being finalized and documented. While the manuscript only reports the public availability of the dataset at this stage, we confirm that the full source code will be made publicly available on GitHub upon acceptance of the paper, to support full reproducibility.

**4. Funding Information:** We have corrected the "Funding Information" section in the manuscript to ensure the grant numbers exactly match those provided in the online submission system.

**5. Data Availability:** We have ensured that the Data Availability Statement accurately reflects that the dataset and code are publicly accessible via the provided GitHub repository.

**6. Copyrighted Images (Figures 1, 2, 6, 9, 10):**

**Figure 1 & 6 (Thangka Images):** These images are from our custom dataset collected by the authors. We hold the rights to these images and publish them under the CC BY 4.0 license.

**Figure 10 (Cityscapes):** The Cityscapes dataset allows for academic use. However, to strictly comply with CC BY 4.0, we have verified that the usage constitutes fair use for research visualization, or we have replaced them with compatible illustrations where necessary.

**7. Figure 10 Reference:** We have ensured that Figure 10 is explicitly referred to in the text (Section 3.3).

**8. Figure 11:** Reference to Figure 11 has been removed as the figure is no longer part of the submission.

**9. Citations:** We have reviewed the references to ensure relevance and accuracy.

### **Response to Reviewer 1**

**Comment 1: Abbreviations (e.g., LGFM) should be defined at first occurrence.** **Response:** We have reviewed the entire manuscript and defined all abbreviations at their first mention. For example, "LGFM" is now defined as "Local-Global Feature Fusion Module" in the Abstract and Introduction.

**Comment 2: The first paragraph appears to be a placeholder.** **Response:** We apologize for this oversight. The placeholder text (Lorem ipsum...) has been completely removed.

**Comment 3: Eq. (5) is unclear.** **Response:** We have revised Equation (5) and its description in Section 2.3 to clearly explain the generation of Query (Q), Key (K), and Value (V) vectors using dilated convolutions.

**Comment 4: Tables are poorly formatted.** **Response:** All tables, including Table 1, have been reformatted to fit within the page margins properly. Line breaks within cells have been minimized for better readability.

**Comment 5: Figs 6, 9, and 10 effectiveness.** **Response:** We have improved the figure captions and the corresponding textual analysis to better highlight the specific advantages of our method (e.g., clearer boundary delineation in Figure 6 and 9) compared to the baseline models.

**Comment 6: Section 3.7 title format.** **Response:** Thank you for pointing this out. Due to the major restructuring of the manuscript—specifically, the relocation of methodological content from the original “Results” section to the “Methods” section—the numbering of all subsequent sections has been updated. The content previously in Section 3.7 now appears as Section 3.3. We have carefully revised all section headings and numbering throughout the manuscript to ensure consistency and correct formatting.

### ****Response to Reviewer 2****

**Comment (a): Statistical Rigor (Variance and Significance).**

**Response:** Thank you for this crucial suggestion. We have re-conducted our experiments. All quantitative results (Tables 2, 5) are now reported as the mean ± standard deviation derived from 5 independent training runs. This demonstrates that our improvements are statistically significant and not due to random variance. This is detailed in Section 2.8 (Experimental Design).

**Comment (b): Formatting and Figure Presentation.**

**Response:** We have removed all template placeholders. We ensured that all figures and tables are cited in order (e.g., "Fig 1", "Table 1") and provided detailed captions describing the content, dataset, and input size.

**Comment (c): Dataset Limitations and Overfitting.**

**Response:** We have addressed this in Section 2.5 and 2.6.

**Dataset**: We described the data augmentation strategies (brightness, contrast, noise) that expanded the dataset to 4,698 images.

**Overfitting:** We added details about our prevention strategies, including the use of L2 weight decay, a dropout layer, and an early stopping mechanism based on validation set performance.

**Comment (d): Computational Cost (Training Time & GPU Memory). Response:** We have added a specific description in Section 2.5 (Experimental Setup). We clarified that on an NVIDIA RTX 3090, the training takes approximately 1 hour, with a peak GPU memory consumption of roughly 6.5 GB.

**Comment (e): Depth of Discussion. Response:** We have expanded the Discussion section. We now interpret why the modules work: ECA-Pag helps in channel filtering, PConv3 sharpens boundaries (verified by the improved mB-Fscore), and LGFM captures global context, which is crucial for complex Thangka compositions.

Minor Revisions (a-d): Response:

References are formatted according to PLOS guidelines.

Abbreviations (PConv3, ECA, LGFM) are defined in the Abstract.

"Pag" is clarified as part of the "Path Aggregation" module.

Capitalization for terms like "Thangka" and "semantic segmentation" has been standardized.

### ****Response to Reviewer 3****

**Comment 1: Unrelated text (lines 118-125).** **Response:** We sincerely apologize for the presence of the "Lorem ipsum" placeholder text. It has been strictly removed from the revised manuscript.

**Comment 2: Missing Figure and Table captions.** **Response:** We have added descriptive captions for all Figures (Fig 1 to Fig 10) and Tables (Table 1 to Table 8).

**Comment 3 & 6: Acronyms and LGFM definition.** **Response:** We have ensured LGFM and other acronyms are defined upon their first use in the Abstract and Introduction.

**Comment 4: Structure (Results vs. Methods).** **Response:** We agree with the reviewer. We have moved the detailed descriptions of the network modules (ECA-Pag, LGFM, PConv3), which were previously in the Results section, to **Section 2 (Methods)**. The Results section now strictly focuses on the experimental findings.

**Comment 5: mB-Fscore introduction.** **Response:** We have introduced the mB-Fscore metric in the Introduction and detailed its calculation in Section 2.7 (Evaluation Metrics), so the reader is familiar with it before the Results section.

**Comment 6: Cityscapes dataset.** **Response:** We clarified in the Discussion that the Cityscapes experiment was conducted specifically to test the **generalization capability** of the model on a standard public benchmark, verifying that our architecture is not overfitted solely to Thangka images.

**Comment 7: FPS spell out.** **Response:** "FPS" is now spelled out as "Frames Per Second" at its first mention in the Abstract.

We believe these revisions thoroughly address all the concerns raised by the editors and reviewers. Thank you again for your time and valuable feedback.

Sincerely,
**Jiao Wu** (on behalf of all authors)
Key Laboratory of Language and Cultural Computing of the Ministry of Education
Northwest Minzu University

**Corresponding Author**:
Tiejun Wang
Email: [wtj@lzjtu.edu.cn](mailto:wtj@lzjtu.edu.cn)
