# Supplementary material for: Research on the semantic segmentation of Thangka images via an improved PIDNet
Source: PLoS One. 2026 May 14;21(5):e0332164. doi: 10.1371/journal.pone.0332164 (PMC13175359; doi:10.1371/journal.pone.0332164)
Supplement: Attachment — Submitted filename: Response_to_Reviewers_auresp_2.docx [file pone.0332164.s005.docx]

**Response to Reviewers**

**Manuscript Title:** Research on the Semantic Segmentation of Thangka Images via an Improved PIDNetAddressed to Reviewer 1 and Reviewer 2

We sincerely thank the two reviewers for their insightful, constructive and detailed comments on our manuscript. These suggestions have been of great help to us in improving the manuscript, and we have carefully addressed all the raised concerns and completed corresponding revisions. The specific responses to each comment and the detailed revision content are as follows:

**Response to Reviewer 1**

**Comment:** Although the authors have addressed my previous concerns, the revised manuscript still contains some writing issues. For example, in Section 3.3, the text shows “Error! Reference source not found.” The authors should carefully review the entire manuscript and correct all writing and formatting errors before the paper can be published.

**Response and Revision:** We sincerely apologize for the writing and formatting errors in the previous version of the manuscript. We have conducted a full and rigorous check and correction of the entire manuscript for all types of writing and formatting issues:

We have fixed the reference source error in Section 3.3 (Experimental Comparison on Cityscapes Dataset), and comprehensively verified the correspondence between all reference citations in the whole manuscript and the References at the end, ensuring that all reference markers are accurate and complete with no missing or mislabeled cases.

We have comprehensively checked the entire manuscript for other formatting errors, including the consistency of figure/table/formula numbering and citation order, the standardization of punctuation and mathematical symbol formatting, the uniformity of unit notation, and the consistency of title level typesetting. All identified errors have been completely corrected.

We have also checked the text for typos and grammatical errors to ensure the accuracy and fluency of the manuscript’s expression.

**Response to Reviewer 2**

**Comment 1**: Data and code availability: Please ensure that the Data Availability Statement in the manuscript clearly matches the information provided in the response letter. If the dataset and code are hosted on GitHub, confirm that the repository is fully accessible and includes all materials necessary to reproduce the experiments (dataset description, training details, and scripts). If the code will only be released upon acceptance, please state this explicitly and clearly in the manuscript.

**Response and Revision:** We have strictly followed your suggestion to revise the Data Availability Statement in the manuscript, and the content in the manuscript is completely consistent with the information in this response letter. We have revised the Data Availability Statement in the manuscript (Section 2.8 Experimental Design) to ensure consistency with this response letter. The Thangka image dataset and the source code of the proposed model will be made fully publicly available at [https://github.com/JikD-591/thangka-seg](https://github.com/JikD-591/thangka-seg) upon the acceptance of this paper. The final repository will include a detailed dataset description, complete training/validation/test scripts, pre-trained model weights, and experimental configuration files, which are sufficient to reproduce all the experimental results reported in the paper. At present, the dataset has been partially uploaded to the repository (split into 4 compressed packages due to platform file size limitations), and the full source code and related experimental files will be supplemented immediately after the paper is accepted. The updated Data Availability Statement in the manuscript clearly and explicitly states the release time of the dataset and code, the repository address, and the specific materials included in the final repository to ensure the reproducibility of all experiments.

**Comment 2:** Minor formatting and consistency: Please conduct a final careful check of the manuscript for any remaining formatting inconsistencies, duplicated sentences (particularly in the Methods section), and figure/table references. Ensure that all abbreviations are defined at first use and that all figures and tables are cited in order within the text.

**Response and Revision:** We have completed a comprehensive and final check of the entire manuscript to ensure the consistency of formatting and the accuracy of content, and the key revisions are as follows:

Formatting consistency: We have unified the formatting of the entire manuscript, including title levels, table/figure caption descriptions, formula numbering and symbol expression, and eliminated all formatting inconsistencies. For duplicated sentences in the Methods section (such as repeated training configuration descriptions), we have deleted the redundancies and streamlined the content.

Figure/table references: We have verified all figure (Fig.1-10) and table (Table1-8) citations in the manuscript, and ensured that all figures and tables are cited in the exact order of their appearance in the text, with no missing, out-of-order or mislabeled citations.

Abbreviation definition: We have checked all English abbreviations in the manuscript (e.g., ECA-Pag, LGFM, PConv3, mIoU, mB-Fscore, FPS, Dconv, PPM), and strictly ensured that all abbreviations are fully spelled out and clearly defined at their first use in the text, with consistent and standardized usage in the subsequent content.

**Comment 3:** Clarity of presentation: Although the manuscript is now much clearer, a final round of language polishing and concise wording in certain sections (especially Methods and Discussion) would improve readability and flow. This is only an editorial refinement and does not require additional experiments or analyses.

**Response and Revision:** We have conducted a comprehensive language polishing and content streamlining for the key sections of the manuscript:

Methods section: We have streamlined redundant technical descriptions of each module, split overly long and complex sentences into concise and clear ones, strengthened the logical connection between paragraphs with appropriate transition words, and made the technical expression more accurate and the logical structure more clear.

Discussion section: We have deleted redundant experimental result descriptions (excluding the core conclusion of cross-domain generalization), compressed verbose modifying statements, and focused on the core research contributions, limitations of the study and future research directions, making the discussion more concise, focused and in-depth.

Section 3.2 (Ablation Experiments): We have deleted the redundant descriptions of module functions (the detailed module principles are only retained in Sections 2.2-2.4), and focused on the analysis of experimental results and data. All conclusions are closely combined with specific experimental indicators (e.g., mIoU/mB-Fscore improvement, FPS changes, category-level IoU/B-Fscore gains), which strengthens the correlation between experimental results and research conclusions.

In addition, we have also polished the language of other sections of the manuscript appropriately, eliminated awkward expressions and redundant sentences, and further improved the overall readability and logical flow of the manuscript.

We would like to express our sincere gratitude to you again for your valuable time and constructive suggestions.

**Sincerely,**

**Jiao Wu** (on behalf of all authors)

Key Laboratory of Language and Cultural Computing of the Ministry of Education
Northwest Minzu University

**Corresponding Author**:

Tiejun Wang

Email: [wtj@lzjtu.edu.cn](mailto:wtj@lzjtu.edu.cn)
